# Uncovering the dominant contribution of intermediate volatility compounds in secondary organic aerosol formation from biomass-burning emissions

**DOI:** 10.1093/nsr/nwae014

**Published:** 2024-01-09

**Authors:** Kun Li, Jun Zhang, David M Bell, Tiantian Wang, Houssni Lamkaddam, Tianqu Cui, Lu Qi, Mihnea Surdu, Dongyu Wang, Lin Du, Imad El Haddad, Jay G Slowik, Andre S H Prevot

**Affiliations:** Environment Research Institute, Shandong University, Qingdao 266237, China; Laboratory of Atmospheric Chemistry, Paul Scherrer Institute, Villigen 5232, Switzerland; Laboratory of Atmospheric Chemistry, Paul Scherrer Institute, Villigen 5232, Switzerland; Laboratory of Atmospheric Chemistry, Paul Scherrer Institute, Villigen 5232, Switzerland; Laboratory of Atmospheric Chemistry, Paul Scherrer Institute, Villigen 5232, Switzerland; Laboratory of Atmospheric Chemistry, Paul Scherrer Institute, Villigen 5232, Switzerland; Laboratory of Atmospheric Chemistry, Paul Scherrer Institute, Villigen 5232, Switzerland; Laboratory of Atmospheric Chemistry, Paul Scherrer Institute, Villigen 5232, Switzerland; Laboratory of Atmospheric Chemistry, Paul Scherrer Institute, Villigen 5232, Switzerland; Laboratory of Atmospheric Chemistry, Paul Scherrer Institute, Villigen 5232, Switzerland; Environment Research Institute, Shandong University, Qingdao 266237, China; Laboratory of Atmospheric Chemistry, Paul Scherrer Institute, Villigen 5232, Switzerland; Laboratory of Atmospheric Chemistry, Paul Scherrer Institute, Villigen 5232, Switzerland; Laboratory of Atmospheric Chemistry, Paul Scherrer Institute, Villigen 5232, Switzerland

**Keywords:** atmospheric chemistry, biomass burning, secondary organic aerosol, intermediate volatility organic compounds

## Abstract

Organic vapors from biomass burning are a major source of secondary organic aerosols (SOAs). Previous smog chamber studies found that the SOA contributors in biomass-burning emissions are mainly volatile organic compounds (VOCs). While intermediate volatility organic compounds (IVOCs) are efficient SOA precursors and contribute a considerable fraction of biomass-burning emissions, their contribution to SOA formation has not been directly observed. Here, by deploying a newly-developed oxidation flow reactor to study SOA formation from wood burning, we find that IVOCs can contribute ∼70% of the formed SOA, i.e. >2 times more than VOCs. This previously missing SOA fraction is interpreted to be due to the high wall losses of semi-volatile oxidation products of IVOCs in smog chambers. The finding in this study reveals that SOA production from biomass burning is much higher than previously thought, and highlights the urgent need for more research on the IVOCs from biomass burning and potentially other emission sources.

## Teaser

By minimizing wall losses of oxidation products, it is found that intermediate volatility organic compounds are the dominant precursors of secondary organic aerosol in biomass-burning emissions.

## INTRODUCTION

Biomass burning, including open and indoor biofuel combustion, is one of the largest sources of atmospheric aerosols and trace gases [1], and therefore dramatically impacts air quality, global climate, and human health [2,3]. Wildfires and other open burning contribute to >50% of the global black carbon and primary organic aerosol, and some megafires can deplete the stratospheric ozone layer [4,5] and enhance cloud formation [6,7]. Wildfire emissions can transport for a long distance (e.g. from the US to Europe) [8], making them ubiquitous even in the remote troposphere [9]. Due to climate change, wildfires have increased in frequency, intensity, and area in the past decades, thus becoming a more and more important emission source of air pollutants [10–12]. On the other hand, residential wood combustion, although not such a major global emission source as wildfires, also significantly impacts regional air quality due to its wide application for heating in winter [3,13–15]. For example, it can contribute up to ∼70% of the organic aerosol and ∼50% of the non-methane organic gases (NMOGs) in Europe in winter [14,15].

When transported in the atmosphere, NMOGs from biomass burning can be oxidized and condensed into the particle phase to form secondary organic aerosols (SOAs). Biomass burning is the second largest emission source of NMOGs worldwide, therefore significantly contributing to SOA formation [1,11]. Based on volatility, NMOGs are divided into several categories: volatile organic compounds (VOCs) with saturation concentration (C*) >3 × 10^6^ μg m^−3^, intermediate volatility organic compounds (IVOCs) with C* in the range of 3 × 10^2^ μg m^−3^ to 3 × 10^6^ μg m^−3^, and semi-volatile organic compounds (SVOCs) with C* in the range of 3 × 10^−1^ μg m^−3^ to 3 × 10^2^ μg m^−3^ [16]. Previous studies have found that IVOCs and SVOCs are a large source of SOA [17,18] and can contribute to a significant fraction of SOA in some complex emissions, such as oil evaporation [19,20] and vehicle exhaust [21].

SOA from biomass-burning emissions has been extensively investigated in laboratory studies in the past few decades, mainly using smog chambers [11,22–26]. These studies identified the most important SOA precursors to be a few NMOG classes: aromatic hydrocarbons, monoterpenes, oxygenated aromatics, and heterocyclic compounds (most of them are VOCs) [23–25]. In other words, SOA production from biomass-burning emissions was thought to be mainly dominated by VOCs [23–25]. However, recent studies reveal that there are considerable IVOCs and SVOCs from biomass-burning emissions [27,28]. These lower-volatility compounds generally have higher SOA yields than VOCs [29–31], but they were not previously found to contribute much to SOA production in smog chambers. A possible explanation for this omission is the high vapor wall losses in smog chambers [32,33]. The vapor wall losses can be corrected by conducting a series of experiments with different seed concentrations [32], but the results are highly dependent on several parameters [33] and thus introduce a large uncertainty.

Here, using a state-of-the-art laminar-flow oxidation reactor (LFOR, see Methods), we present the first direct evidence of the significant contribution of IVOCs to SOA production from biomass-burning emissions. The LFOR minimizes the wall losses of semi-volatile vapors and particles [31] and is a powerful tool to evaluate the contributions of different volatility categories of organic vapors to SOA production. The SOA closure was evaluated by comparing measured aerosol mass concentrations and calculated aerosol mass concentrations from SOA yields and NMOG concentrations measured with a proton transfer reaction time-of-flight mass spectrometer (PTR-TOF-MS, hereinafter referred to as PTR-MS), similar to the method of Bruns *et al.* [23]. We also deployed a Vocus PTR-TOF [34] (hereinafter referred to as Vocus-PTR) to measure IVOCs from biomass-burning emissions. In addition, we used an extractive electrospray ionization time-of-flight mass spectrometer (EESI-TOF-MS, hereinafter referred to as EESI) [35] to measure molecular-level aerosol composition. With the combination of these cutting-edge techniques, we are able to quantify the contribution of IVOCs to SOA in wood-burning emissions. The results of this study greatly improve our understanding of the impacts of biomass burning on the global aerosol burden and corresponding environmental and climate effects.

## RESULTS AND DISCUSSION

### SOA formation

**Figure 1.**
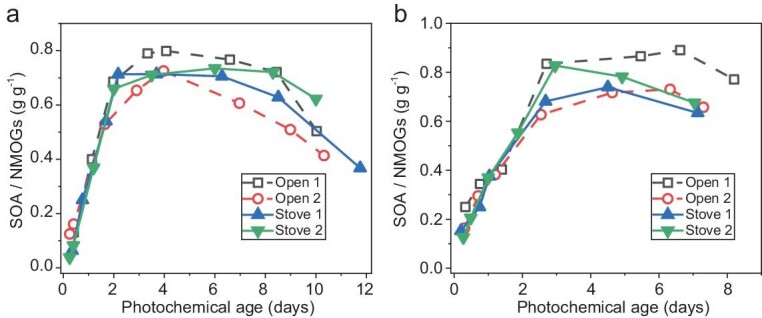
SOA production normalized by total NMOG concentration measured with PTR-MS. (a) Low-NO_x_ condition; (b) high-NO_x_ condition. Note that only compounds with carbon number ≥4 were considered since smaller compounds have negligible SOA yields. The equivalent photochemical age was calculated by dividing the OH exposure in the LFOR by the global average OH concentration of 1.5 × 10^6^ molecules cm^−3^.

Pine and spruce woods were burned open or in a stove to mimic wildfires or residential wood combustion and the emissions were introduced into the LFOR (Fig. S1 and Table S1 in the supplementary materials online). The oxidation experiments in the LFOR were performed under both low- and high-NO_x_ conditions to simulate the different oxidation regimes in the atmosphere (see Methods and Text S1 in the supplementary materials online). SOA formation from open and stove burning under low- and high-NO_x_ conditions in the LFOR is shown in Fig. S2 as a function of photochemical age. There are some variations among different experiments, likely from the difference in NMOG concentrations. Therefore, the SOA formation was normalized by the total mass concentration of NMOGs with carbon number ≥4 that was measured with PTR-MS (smaller NMOGs were considered to contribute little to SOA formation), as shown in Fig. 1.

After normalization, the maximum SOA formation potential (in terms of g g^−1^ NMOGs) is ∼0.7–0.8, which is very similar for both open and stove burning, and for both low- and high-NO_x_ conditions. Although the modified combustion efficiency (MCE, defined as ΔCO_2_/(ΔCO+ΔCO_2_), see Table S1) is in a relatively narrow range (>0.9, indicating mostly flaming burn phase), the results in Fig. 1 suggest that SOA formation from the same wood type is likely independent of the burning method (i.e. open or stove burning) or the NO_x_ conditions. Previous studies showed that different burning phases (i.e. flaming or smoldering) have a large influence on the emission factors (EFs) of particles and NMOGs [26,36–38]. We also observe large variations in EFs for individual burns (see Table S1). However, it mainly affects the absolute intensities of NMOGs, but influences little the relative SOA potential of NMOGs, at least for flaming emissions. The results here highlight the same SOA formation potential (after normalized by NMOGs) for wood-burning emissions with various burning and NO_x_ conditions.

### VOCs and IVOCs contributions to SOA

The contribution of NMOGs (measured with PTR-MS) to SOA production was calculated to better understand the SOA precursors (see Methods and Text S2 for details). With the original chamber SOA yield (Table S2), the SOA formation and contribution from NMOGs measured with PTR-MS are estimated and are shown in Fig. S3. It is shown that the contribution of these NMOGs to measured SOA decreases with increasing photochemical age, especially at the range of <2 days. This is likely due to the different SOA yield at different photochemical ages in the LFOR [31,39]. Therefore, the scaled yields were applied to the LFOR dataset. Briefly, we use 5 representative NMOGs to get a reference pattern of the change in SOA yield at different photochemical ages, and use this pattern to scale the smog chamber (SC) yields to the LFOR experiments. Further details regarding this correction are shown in Text S3 and Fig. S4.

**Figure 2.**
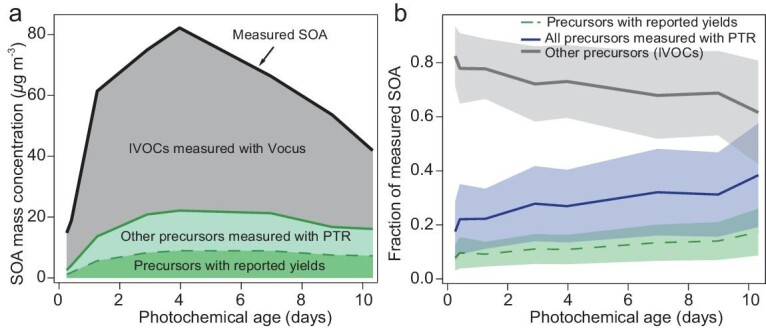
SOA production and contribution from NMOGs. (a) Measured SOA mass concentration and calculated SOA mass concentration from the consumption of NMOGs measured with PTR-MS. (b) Contribution of NMOGs to SOA production. The data are taken from the open-low-2 experiment as an example. The SC-LFOR yield correction (see Text S3 and Fig. S4) was applied to all precursors measured with PTR-MS. For precursors with reported SOA yields, the yields can be found in Table S2.

With the scaled yields, the SOA formation and contribution from NMOGs measured with PTR-MS in a representative experiment (open burning low-NO_x_ experiment 2) are shown in Fig. 2. The contributions of NMOGs to SOA in Fig. 2 are relatively stable under different photochemical ages. The overall contribution of NMOGs with reported yields in this experiment is 13 ± 6%, while the overall contribution of all NMOGs measured with PTR-MS is 28 ± 14% (see Text S4 for uncertainty estimation). All LFOR experiments show similar results with this representative experiment, with an average of 27 ± 14% for the contribution of all NMOGs measured with PTR-MS (Fig. S5). This low contribution (∼27%) is different from the previous SC data with stove burning of beech wood [23] and open burning of western US fuels [25] showing that NMOGs measured with PTR-MS can explain all the observed SOA. To investigate if this is due to different burning phase (i.e. flaming or smoldering) or wood type (i.e. hardwood vs softwood), we also performed smog chamber experiments with a similar burning setup as the LFOR experiments (see Text S1 for details). As shown in Fig. S6, these smog chamber experiments get good SOA closure similar to previous studies [23,25], which excludes the influence of different burning setups.

The missing SOA source in the LFOR is interpreted to be from the oxidation products of lower-volatility compounds (e.g. IVOCs and/or SVOCs). These oxidation products are lost to walls in smog chambers but proceed to produce SOA in the LFOR. To verify this, a Vocus-PTR was deployed in a subset of experiments to quantify IVOCs. Although the Vocus-PTR may not measure all NMOGs, it has been previously found that it can measure organic vapors with a wider range of volatilities compared to conventional PTR-MS [40]. The NMOGs classified by volatility measured by both the PTR-MS and Vocus-PTR are shown in Fig. S7. For the PTR-MS, only ∼3% of the measured NMOGs are IVOCs. In contrast, the fraction of IVOCs measured by Vocus-PTR is 12.9% (in the LFOR), indicating that most of the IVOCs are not fully quantified with the PTR-MS, as demonstrated in a previous intercomparison study [40]. As shown in Fig. S8, the difference is partially from the same compounds measured with both the PTR-MS and Vocus-PTR while the Vocus-PTR generally measures higher concentrations; for lower volatility (C* <1 × 10^4^ μg m^−3^) compounds with higher oxygen number (≥3), only the Vocus-PTR has the capability to measure them [40]. In absolute terms the concentrations of measured VOCs with the PTR-MS and Vocus-PTR are the same, while the Vocus-PTR measures 11.4% more IVOCs (of all NMOGs) compared to PTR-MS. The interpretation is that the difference in IVOCs contributes to the majority of the missing SOA (i.e. ∼70% of the measured SOA), according to the analysis below.

### Evidence on IVOC contribution to SOA

**Figure 3.**
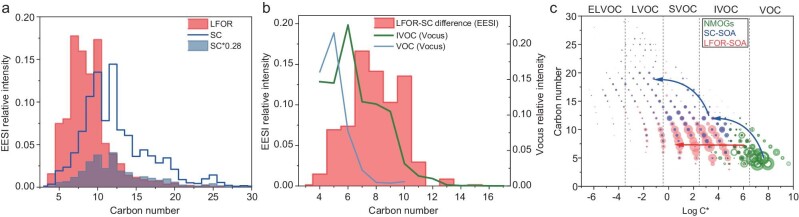
SOA and NMOG composition from biomass burning. (a) SOA composition (in terms of carbon-number distribution) measured with EESI in the LFOR and SC experiments at photochemical age of ∼2 days. (b) Carbon-number distribution of the difference in SOA formed in the LFOR and SC experiments (measured with EESI) and IVOC and VOC (measured with Vocus-PTR). (c) Carbon number versus volatility for NMOGs and SOA formed in the SC and LFOR. The smog chamber data was scaled with a factor of 0.28. The blue and red arrows refer to the possible evolution pathways for VOCs and IVOCs, respectively.

The SOA composition (in terms of carbon-number distribution) measured with EESI for the LFOR and SC experiments is shown in Fig. 3a. When treating the intensity of SOA from SC and LFOR the same (i.e. as 1), it is found that SOA in SC experiments has more high-carbon-number compounds (*n_C_* >10) than SOA in LFOR experiments. However, the SOA from SC only accounts for ∼28% of the SOA from LFOR (Fig. 2b). Therefore, a scaling factor of 0.28 is applied to the SC SOA. After scaling, the SC SOA agrees well with the LFOR SOA for compounds with *n_C_* ≥12. The differences are mainly C_5_–C_10_ compounds, which have similar carbon-number distribution with the IVOCs rather than VOCs (Fig. 3b). This suggests that the difference in the SC and LFOR SOA (i.e. ∼70% of the LFOR SOA) is likely to be from IVOCs that can be measured with Vocus-PTR.

It is also noticed that although VOCs have lower carbon numbers compared to IVOCs (Fig. 3b), SOAs formed from them tend to have higher carbon number than SOAs formed from IVOCs (Fig. 3a). This may be explained by the following reason. Lower-carbon-number VOCs have high volatilities, so simply adding oxygen to the molecules cannot efficiently form products that can condense into particle-phase. Therefore, more dimers, trimers and other oligomers are formed and are present in the SOA [41–43], leading to the ‘jump’ in carbon number and volatility (Fig. 3c). With the ‘jump’ in carbon number and the corresponding decrease in volatility through oligomerization, VOCs can form products with volatilities low enough to generate particles rather than lost on the wall. However, IVOCs have higher carbon numbers and lower volatilities, so a few oxidation steps can form products with volatilities low enough to condense (Fig. 3c). When IVOCs are slightly oxidized, they form products readily lost on the wall but not efficiently generate particles in the SC (since the chamber wall has much more surface area than the aerosols), e.g. many SVOCs have a lifetime of ∼10 min due to the high wall loss rate [44]. In contrast, the wall loss rates of semi-volatile compounds (e.g. H_2_SO_4_) in the LFOR are minimal [31]. This is likely the reason that different SOA formations and carbon-number distributions are observed in the SC and LFOR experiments.

To verify this hypothesis, we estimate the fates of organic vapors with a simplified model using the KinSim kinetic integrator [33,45]. At first, we only consider the physical processes in the smog chamber and the LFOR including condensation (to wall and particles) and evaporation for LVOCs and SVOCs (see Text S5.1 for details). As shown in Fig. S9, the LVOC fates in the SC and the LFOR are quite similar: >97% of them are in the particle phase at the end of the experiments. However, the SVOC fates in the SC and the LFOR are quite different. In the SC, only ∼16% of the SVOCs are in the particle phase, while ∼80% of the SVOCs are lost on the wall due to the high wall loss rate and long reaction time. In the LFOR, ∼80% of the SVOCs are in the particle phase, while only ∼3% are lost on the wall.

**Figure 4.**
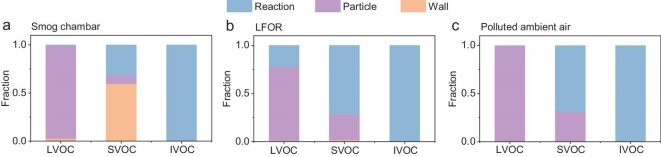
Estimated fates of LVOC, SVOC, and IVOC in (a) smog chamber, (b) LFOR, and (c) polluted ambient air. Note that LVOC, SVOC, and IVOC are assumed to have C* of 0.001 μg m^−3^, 10 μg m^−3^, and 100 000 μg m^−3^, respectively. More details about this estimation can be found in Text S5 and Fig. S10.

Furthermore, we estimate the fates of IVOCs, SVOCs, and LVOCs in the SC, in the LFOR, and in the polluted ambient air with the consideration of OH reactions (Text S5.2, Fig. 4 and Fig. S10). As shown in Fig. 4, the main fate of IVOCs is the reaction with OH (>99%) while the main fate of LVOCs is condensation to be particles (>80%) for all three scenarios. However, the fates of SVOCs are quite different. There are two main fates for SVOCs in the LFOR and in the ambient air: reaction with OH (∼70%) and condensation onto particles (∼30%). However, the main fate of SVOCs in the SC is wall loss (∼60%), while reaction and condensation contribute to smaller fractions (∼31% and ∼9%, respectively). This explains well the SOA mass concentration and composition differences observed in the SC and the LFOR. The results here indicate that the high wall loss rates (especially for SVOCs) are a weakness of the SC for simulating processes in the real atmosphere [33], while the LFOR can overcome this weakness.

**Figure 5.**
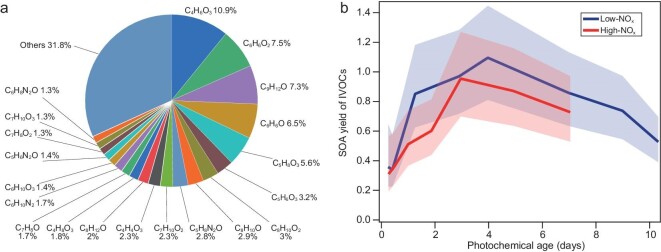
Biomass burning IVOC composition and SOA yield. (a) IVOC compounds that contribute to the SOA difference in the LFOR and SC experiments. The top 20 species are shown, while other compounds contribute 31.8% of total IVOC. (b) Average SOA yield of IVOCs under low- and high-NO_x_ conditions.

In addition, we assess the hypothesis by looking into the molecular composition of SOAs. Fig. S11 shows the relative intensities of representative products measured with EESI from the oxidation of phenol (C_6_H_6_O) and dimethylphenol (C_8_H_10_O), which are two typical IVOCs that contribute to the difference in SOA production in LFOR and SC (Fig. 5a). These data are from the same condition as Fig. 3a, i.e. at a photochemical age of ∼2 days. It is shown that the intensities of C_6_ products from phenol (C_6_H_6_O_3_, C_6_H_6_O_4_, C_6_H_6_O_5_, C_6_H_8_O_6_, and C_6_H_8_O_7_) [46,47] and C_8_ products from dimethylphenol (C_8_H_10_O_5_, C_8_H_12_O_6_, C_8_H_10_O_7_, and C_8_H_12_O_7_) [46] in the LFOR are significantly higher than those in the SC by at least an order of magnitude. This indicates that these products and/or the intermediate species that lead to the formation of these products may have strong wall losses in the SC but little wall losses in the LFOR, which further supports our hypothesis above.

### Average SOA yield of IVOCs

The composition of IVOC difference measured with Vocus-PTR and PTR-MS in the wood-burning emission experiment is shown in Fig. 5a. The top 20 species are mainly C_4_–C_9_ compounds containing 1–3 oxygen atoms, and contribute ∼70% in concentration. With the chemical information, we calculated the overall SOA yields of IVOCs (see Text S6 for details), as shown in Fig. 5b. The SOA yields of IVOCs are similar in high- and low-NO_x_ experiments, which agrees well with the overall SOA production from NMOGs (Fig. 1). The maximum SOA yield is about 1–1.1, which is higher than most VOCs in the LFOR (<0.6, from a previous study [31] and Table S3). It is also higher than the yield of some IVOCs, e.g. *n*-dodecane (∼0.8) [31] and *o*-cresol (∼0.7, see Table S3), and is similar to that of naphthalene (∼1, see Table S3). It should be noted that the SOA yield of some IVOCs can be up to 1.5–2 (e.g. alkanes with one or more rings) [31,48]. The average yield of VOCs in this study was also estimated, which has a maximum of ∼0.4, about 2.5 times lower than that of IVOCs. Therefore, the derived SOA yield of biomass-burning IVOCs (up to ∼1) is likely in a reasonable range and might be applied to the parameterization of SOA estimation in field studies.

### Implications

This study highlights the importance of IVOCs in the SOA formation from biomass burning, which remains poorly understood. To our knowledge, this is the first direct evidence indicating that IVOCs contribute much higher than VOCs to SOA in biomass-burning emissions. The results here suggest that more research on the IVOCs is needed for wood burning and other biomass-burning emissions, which can be done with cutting-edge (e.g. Vocus-PTR) and more advanced techniques.

The results here also indicate that using a smog chamber as the tool to study SOA formation may largely underestimate the contribution of lower-volatility compounds (e.g. IVOCs and their oxidation products, SVOCs) in complex emissions. Thus, the results of previous smog chamber studies for complex emissions (e.g. biomass burning, oil evaporation, vehicle exhaust, etc.) need to be used with caution and may need to be revisited with state-of-the-art oxidation flow reactors with minimal wall losses, e.g. LFOR.

Last, the SOA yield of IVOCs from biomass burning obtained in this study may greatly improve the parameterization of SOA production in field studies. With the emission inventory or measured total concentration of IVOCs from biomass burning, one could estimate their SOA production, which improves our understanding of SOA formation from complex emissions.

## METHODS

### Biomass burning experiments

The experiment setup is shown in Fig. S1. Pine and spruce woods (0.5–1.2 kg) were burned open and in a stove to mimic forest fire and residential wood combustion, respectively. Barks, twigs, and needle leaves were used for the open burning as they are the main parts that are burned in forest fires. Logwoods were used in stove burning, similar to previous studies [22,23,26]. The burning emissions were injected into a 1 m^3^ stainless steel holding tank or an 8 m^3^ smog chamber with an ejection diluter (DI-1000, Dekati Ltd.). During the injection, the concentrations of CO, CO_2_, total hydrocarbon (THC), and particles were measured *in situ*. For most of the experiments, the MCE was in the range of 0.9–0.95 (see Table S1), indicating a flaming burning phase slightly mixed with the smoldering burning phase [11,49]. Further details regarding the biomass burning experiments are provided in Text S1.

### Flow reactor

After the emission concentration reached a considerable level (THC >5 ppm), the injection stopped and the gas in the holding tank was introduced into an oxidation flow reactor (OFR). The details of the OFR were described previously [31,39,50]. Briefly, it has a different design compared to most previous OFRs, involving improved fluid dynamics leading to much smaller wall losses [31]. This OFR is named laminar-flow oxidation reactor (LFOR) since it can minimize jetting and recirculation in the reactor and makes the flow more laminar [31]. In LFOR, both low- and high-NO_x_ OH oxidation experiments were performed for the wood-burning emissions. The high-NO_x_ experiments were realized by introducing %-level N_2_O, see previous studies for details [39,51]. The RO_2_ radicals predominately react with HO_2_ in low-NO_x_ experiments, while ∼90% of the RO_2_ radicals react with NO in high-NO_x_ experiments. A series of recent modeling studies demonstrate that the chemistry in OFR can be similar to the troposphere when several conditions are carefully controlled [52]. We followed these instructions by using low precursor concentrations, relatively high humidity, and long residence time to realize the chemistry in the LFOR close to the atmospheric OH oxidation. More details on the LFOR are provided in Text S1, and the potential limitations of OFRs are discussed in Text S7.

### Measurements and calculations

CO and CO_2_ were measured with gas analyzers (Horiba APMA-370 and LI-COR LI-7000). The concentrations of THC and methane were monitored by a flame ionization detector monitor (Horiba APHA-370). The gas-phase organic compounds were measured with a PTR-TOF-MS (Ionicon). For a subset of experiments, a Vocus PTR-TOF (Tofwerk) [34] was deployed to measure organic vapors with a wider range of volatilities (compared to conventional PTR-MS) [40]. The decay of NMOGs in the holding tank was 10%–15% in 5–6 h (the duration of an LFOR experiment) according to Vocus-PTR measurements and was the same for VOCs and IVOCs. The OH exposure in the LFOR was estimated by the decay of benzene and toluene measured with PTR-MS. The equivalent photochemical age was calculated using a global average OH concentration of 1.5 × 10^6^ molecules cm^−3^ [53].

Aerosols were measured with a scanning mobility particle sizer (SMPS, TSI), a long time-of-flight aerosol mass spectrometer (LTOF-AMS, Aerodyne), and an EESI-TOF-MS [35]. The SOA closure was calculated from the NMOGs measured with PTR-MS and their SOA yields, similar to a previous study [23]. The SOA yields were taken from previous smog chamber studies [29,46,47,54–62], see Table S2. Both original and scaled yields were used to calculate the SOA production (see Texts S2 and S3). For NMOGs with carbon number (*n_C_*) ≥6 and without reported SOA yield, the SOA yields were determined from the average of applied yields for *n_C_* ≥6 compounds (0.32), similar to Bruns *et al.* [23]. Finally, we assessed the contributions of VOCs and IVOCs to the measured SOA mass concentration. More details on the measurements and calculations can be found in Text S2.

## Supplementary Material

nwae014_Supplemental_File

## Data Availability

The data of this study are available on Zenodo: https://doi.org/10.5281/zenodo.10453642.
