# Supplementary material for: Uncovering the dominant contribution of intermediate volatility compounds in secondary organic aerosol formation from biomass-burning emissions
Source: Natl Sci Rev. 2024 Jan 9;11(3):nwae014. doi: 10.1093/nsr/nwae014 (PMC10883696; doi:10.1093/nsr/nwae014)
Supplement: nwae014_Supplemental_File [file nwae014_supplemental_file.pdf]

Supplementary Information for

Uncovering the dominant contribution of intermediate volatility compounds in secondary organic aerosol formation from biomass-burning emissions

Kun Li[1,2,*], Jun Zhang[2], David M. Bell[2], Tiantian Wang[2], Houssni Lamkaddam[2], Tianqu Cui[2], Lu Qi[2], Mihnea Surdu[2], Dongyu Wang[2], Lin Du[1], Imad El Haddad[2], Jay G. Slowik[2], and Andre S. H. Prevot[2,*]

[1]Environment Research Institute, Shandong University, Qingdao 266237, China.
[2]Laboratory of Atmospheric Chemistry, Paul Scherrer Institute, Villigen 5232, Switzerland.

*Corresponding author: Kun Li, Andre S. H. Prevot
Email: kun.li@sdu.edu.cn, andre.prevot@psi.ch

This PDF file includes:

Texts S1 to S8
Figures S1 to S12
Tables S1 to S5

Supplementary Text

Text S1. Experimental details

As shown in Fig. S1, the biomass-burning emissions were introduced into the holding tank or smog chamber through heated lines (150 °C) with the heated ejection diluter (180 °C). The ejection diluter was operated with an input pressure of ~1.5 bar, resulting in a sheath flow of ~20 L min^{-1} and a central flow of ~2 L min^{-1}. The injecting time to the holding tank was ~10−20 min, while it was ~1−5 min to the smog chamber. During the ejection, CO, CO_2, and THC analyzers were connected to the holding tank or smog chamber to monitor the gas concentrations. After 5−10 min of measurement, the concentrations stabilized and these gas analyzers were disconnected.

Emission factors (*EF*) were calculated using Eq. (S1) adapted from Andreae and Merlet (2001) [1]. For pollutant *i*, P_i (CO, CO_2, THC, PM etc.), the EF can be calculated as:

$$EF_{P_i} = \frac{\Delta P_i}{\Delta C_{CO_2} + \Delta C_{CO} + \Delta C_{THC} + \Delta C_{PM}} \times f_C \tag{S1}$$

where ΔP_i is the background-corrected concentration of pollutant *i*, ΔC_{CO_2}, ΔC_{CO}, ΔC_{THC}, and ΔC_{PM} are the background-corrected carbon concentration of CO_2, CO, THC (including CH_4), and PM, and f_C is the carbon fraction of wood, estimated to be 0.5 [1-3].

The volume of the laminar-flow oxidation reactor (LFOR) is 16 L and the total flow was set to 6 L min^{-1}, resulting in an average residence time of 160 s, which is the same as a previous study [4]. The relative humidity (RH) in the LFOR was controlled at ~50%. The flow from the holding tank to the LFOR was controlled to ~0.1 L min^{-1}, realizing a diluting factor of 60. The ozone concentration in the LFOR was maintained at ~4 ppm by using a constant small flow (0.04 L min^{-1}) through an ozone generator (UV pro). The sampling flow was ~2 L min^{-1} taking from the centre of the LFOR while the rest (~4 L min^{-1}) was exhausted to minimize the wall effects. The OH radicals were produced by the photolysis of O_3 to $O(^1D)$ with Hg lamps (Jelight Inc.), followed by the reaction with water vapor: $O(^1D) + H_2O \rightarrow 2OH$. The OH exposure (i.e., photochemical age) in the LFOR was adjusted by changing the number and input voltage of the Hg lamps [5, 6]. The OH exposure was determined by the decay of benzene and toluene measured with PTR-MS (see Text S2). For high-NO_x experiments, percent-level N_2O was introduced into the LFOR to generate NO: $O(^1D) + N_2O \rightarrow 2NO$ [5, 7]. The RO_2 fate was modelled with OFR Exposures Estimator v3.1 [8]. By using different N_2O concentrations (1%−10%) at various OH exposures, the RO_2 was estimated to react ~90% to NO, with the rest (~10%) mostly react with HO_2, which are typical high-NO_x conditions.

The smog chamber (SC) was located in a temperature-controlled house to maintain a constant temperature (20 °C). A set of 40 × 100 W UV lights (Cleo Performance, Philips) was used for irradiation [9]. The RH was initially set to ~50% when the lights were off. HONO was used as the OH source and was injected by passing a small flow of zero air through the mixture of H_2SO_4 (0.1 mmol L^{-1}) and $NaNO_2$ (0.2 mmol L^{-1}) solution. The d9-butanol was introduced into the chamber for the OH exposure estimation [10]. After the lights were turned on, the RH decreased to about ~40% due to the slight increase in temperature. The lights were on for about 3−5 h, resulting in a maximum photochemical age of 1.5−2.5 days with a global average OH concentration of 1.5 × 10^6 molecules cm^{-3} [11]. The aerosol concentration was wall-loss corrected. Aerosol wall loss rate constant ($k_{w,a}$) in the chamber was determined using the decay of particle mass concentration at the beginning and end of each experiment when lights were off. The wall loss corrected aerosol mass concentration ($Aerosol_{wlc}$) was calculated as:

$$Aerosol_{wlc}(t) = Aerosol_{meas}(t) + \int_0^t k_{w,a} \cdot Aerosol_{meas}(t) \cdot dt \tag{S2}$$

where $Aerosol_{meas}$ is the measured aerosol mass concentration. Vapor wall losses were not corrected because of the following two reasons. First, the wall loss rates of unreacted NMOGs are very low according to our previous studies [2, 12], while the correction of intermediate gas-phase products is highly challenging. Second, almost all the SOA yield data in previous studies (see Text S2 below) used to calculate the SOA production only consider particle wall loss. To get a reasonable SOA closure, vapor wall losses were not counted.

Text S2. SOA and NMOG measurements and calculations

Organic aerosol (OA) mass concentration was calculated by multiplying particle volume concentration (measured with the SMPS) by the particle density (1.6−1.7 g cm^{-3}), which was calculated from the vacuum aerodynamic diameter (obtained from the AMS) and the electric mobility diameter (obtained from the SMPS). The primary OA (POA) mass concentrations

in the LFOR and SC were 15−45 μg m^{-3} and 10−35 μg m^{-3}, respectively. By performing control experiments that remove the NMOGs with denuder, we found that the POA mass concentration change little during oxidation (< 5%). Therefore, the SOA mass concentration was calculated as the difference between total OA mass concentration and the POA mass concentration.

A novel instrument for real-time measurement of organic aerosol composition, extractive electrospray ionization time-of-flight mass spectrometer (EESI-TOF-MS) was deployed for the biomass-burning SOA in this study. EESI can measure aerosol composition without thermal decomposition or ionization-induced fragmentation. The details of the instrument is discussed elsewhere [13] and only briefly overviewed here. Air from LFOR or smog chamber was sampled at 1 L min^{-1}, either directly or through a particle filter to get a background measurement. The flow passed through a multichannel denuder, removing gas-phase species, and then intersected a spray of charged droplets generated by a conventional electrospray capillary. Particles collide with the electrospray droplets and the soluble components are extracted, ionized by Coulomb explosion of the charged droplets, and detected by TOF-MS (mass resolution ~10000). The electrospray working fluid is 100 ppm sodium iodide (NaI) water solution. Spectra are recorded in positive ion mode, in nearly all cases as adducts with Na^+.

SOA production from precursors was mainly estimated using the NMOGs measured with PTR-MS. Although PTR-MS cannot distinguish different isomers and can only provide molecular formulas without identifying the molecular structure, previous studies have identified lots of NMOG structures [12]. As shown in Table S2, we identified 18 NMOGs with reported SOA yields. For other NMOGs with carbon number (n_C) ≥ 6 (with or without an identified structure), the average of applied yields for $n_C \geq 6$ compounds (0.32) was used as their SOA yield. The SOA mass concentration produced from each NMOG was calculated by multiplying the reacted concentration and the SOA yield. The reacted NMOG concentration was calculated by their reaction rate constants with OH (k_{OH}) and the estimated OH exposure. For NMOGs without literature k_{OH} or a molecular structure, the k_{OH} was calculated from molecular formulas using a parameterization method [14]. The volatilities of individual NMOGs with reported structures were taken from literature [15], while those with unknown structure were estimated based upon their molecular formulas using a parametrization described previously [16].

To quantify the NMOGs measured with PTR-MS and Vocus-PTR, the reaction rate constant of each species with the reagent ion (H_3O^+) and a transmission function are needed to convert signal intensity to concentration. When available, individual reaction rate constants from the literature were applied [17] and a default reaction rate constant of 2×10^{-9} cm^3 s^{-1} was applied to all other ions. The transmission function was determined using 12 NMOGs in a gas standard (acetaldehyde, methanol, acetonitrile, acetone, acrylonitrile, isoprene, methyl ethyl ketone, benzene, toluene, m-xylene, α-pinene, 1,2,4-trimethylbenzene).

Text S3. Correction between LFOR and smog chamber SOA yields

As the SOA yields from LFOR experiments are largely limited and there are more available SOA yields data from smog chambers, we had to use the reported smog chamber SOA yields for the SOA production calculation in this study. However, it was noticed previously that the SOA yield changed at different photochemical ages in the LFOR [6, 18], while the chamber SOA yields are mostly at <2 photochemical days. Therefore, a correction is needed to apply this yield to a wider range of photochemical ages.

The idea is to perform LFOR experiments with several single NMOGs (the representatives in the biomass-burning emissions), and get the SOA production pattern under different photochemical ages. The pattern (normalized SOA yield curve) is then used to correct the difference observed under low (~1 day) and high (up to ~14 days) photochemical ages. We chose 5 NMOGs to perform the LFOR experiments, including 3 VOCs (benzene, toluene, and *α*-pinene) and 2 IVOCs (*o*-cresol and naphthalene), which are among the most important contributors to SOA production from the wood burning according to PTR-MS measurements in this study. These NMOGs were introduced into the LFOR by using a small flow passing on top of the glass vial with liquid/solid chemicals, reaching a concentration of 10-50 ppb (see Table S3 and Fig. S4a). The SOA yields normalized by the maximum were put together and were fitted to get a curve (Fig. S4b). As smog chamber yields were mostly obtained at the photochemical age of ~1 day, this yield curve was divided by the value at 1 day (0.52) to get a correction factor curve (Fig. S4c). This correction factor curve was used to correct all the SOA production from NMOGs in the LFOR.

Text S4. Estimation of uncertainty in the contribution of measured NMOGs to SOA

The overall uncertainty associated with the PTR-MS-NMOG contribution to SOA contains the uncertainties from the following three parts.

(1) SOA mass concentration. SOA mass concentration was calculated by volume concentration and particle density, which has a small uncertainty of ±10%.

(2) NMOG concentration. The uncertainties in PTR-MS measurements are mainly from: a. uncertainty in reaction rate constant with H_3O^+; b. uncertainty in transmission efficiency. According to previous studies [4, 19], the overall uncertainty in PTR-MS measurement is ±30%.

(3) SOA yield.

a. The uncertainty in original chamber yield of individual precursor is estimated as ±50%.

b. The uncertainty in the scaling factor between SC and LFOR. When using the 5 NMOGs to get a fitting curve, an uncertainty of 0.15 in the normalized yields is estimated (Fig. S4b). This leads to 30−40% uncertainty in the applied factor (depends on photochemical age, see Fig. S4c). We use ±40% for the estimation below.

The uncertainty in applied SOA yield from individual NMOGs (i.e., yield (±50%) × factor (±40%)) is ±64%. When summing them up, the error of each NMOG is uncorrelated [19], so the summed uncertainty for these errors is estimated as the quadrature addition of each absolute uncertainty. This leads to an uncertainty in overall SOA yield to be 15−20%.

Overall, the uncertainty in NMOG concentration is ±30%, which is mainly from the calibration and can be a systematic error. Therefore, the SOA production (NMOG concentration × SOA yield) uncertainty is estimated by directly adding the two errors together, which is 45−50%. After considering errors in SOA mass concentration, the overall uncertainty changes very little, so we estimate the overall uncertainty in the contribution to be ±50%.

When photochemical age is shorter than 2 days, the uncertainty in reaction rate constant with OH (k_{OH}) can also influence the overall uncertainty. The uncertainty of 40% k_{OH} is assumed and accounted in the results shown in Fig. 2b.

Text S5. Kinetic model of organic vapor fates

S5.1 LVOC and SVOC fates without considering reactions

The fates of lower-volatility organic vapors (i.e., LVOC and SVOC) in the smog chamber and LFOR is modeled with the KinSim (v. 4.1.6) chemical kinetic integrator [20] to provide a simplified understanding of how these vapors evolve in different reactors. In this part, we follow the framework of Krechmer et al. [21] but remove the chemical transformation, i.e., only the condensation on aerosol particles and wall and the evaporation are considered. The parameters applied in the model are listed in Table S5. The initial organic aerosol mass concentration (C_{OA}) is set to 30 μg m^{-3}, which is the typical POA concentration used in our smog chamber and LFOR experiments. The initial concentrations of SVOC and LVOC are both set to 1 ppb, and no formation sources are presented during the simulation (i.e., precursor concentration is set to 0). The saturation mass concentrations (C*) of SVOC and LVOC are assumed to be 10 μg m^{-3} and 0.001 μg m^{-3}, respectively.

Both SVOC and LVOC condense on the preexisting organic aerosol particles and wall. The condensation rate onto the particles ($k_{cond,p}$) is calculated based on the suspended aerosol condensation sink (CS) [6, 22]:

$$k_{cond,p} = 4\pi \, CS \, D \tag{S3}$$

where D is the diffusion coefficient, which is estimated to be 6 × 10^{-6} m^2 s^{-1} [21, 23]. The model calculates a CS at each time step based on the total amount of aerosol present at each point in the simulation using the following equation:

$$CS = \int_0^\infty r \, F_{FS}(r) \, N(r) \, dr \tag{S4}$$

where r is the particle radius, $N(r)$ is the particle number size distribution, and F_{FS} is the Fuchs−Sutugin correction for gas-phase diffusion [21, 22, 24]:

$$F_{FS} = \frac{Kn + 1}{0.377Kn + 1 + \frac{4}{3}\alpha^{-1}Kn^2 + \frac{4}{3}\alpha^{-1}Kn} \tag{S5}$$

where α is the mass accommodation coefficient (also known as the sticking coefficient) of condensing vapor, which is assumed to be 1 [25]. *Kn* is the Knudsen number:

$$Kn = 3\sqrt{\frac{\pi M}{8RT}}\frac{D}{r} \tag{S6}$$

where M is the molecular weight of the condensing vapor, which is assumed to be 200 g mol^{-1}; R is the gas constant; T is the temperature.

We assume that SVOC re-evaporates from particles and wall but LVOC does not, since the C^* of LVOC is very low. The evaporation rate of SVOC from particles ($k_{evap,p}$) is calculated from $k_{cond,p}$ and equilibrium constant as:

$$k_{evap,p} = k_{cond,p}\frac{C^*}{C_{OA}} \tag{S7}$$

In the smog chamber, the condensation rate of organic vapor onto the wall ($k_{cond,w}$) is estimated to be 1.7 × 10^{-3} s^{-1}, corresponding to a lifetime of ~10 min, which is the typical timescale for SVOC and LVOC [26, 27]. The evaporation rate of SVOC from the wall ($k_{evap,w}$) is calculated from $k_{cond,w}$ and equilibrium constant as:

$$k_{evap,w} = k_{cond,w}\frac{C^*}{C_w} \tag{S8}$$

where C_w is the equivalent wall mass concentration, expressed as the equivalent amount of organic aerosol particles that would result in the same gas-wall equilibrium condition [28]. Follow Krechmer et al. [27], we calculate the C_w of SVOC to be 63.7 μg m^{-3} by:

$$C_w = 16\,(C^*)^{0.6} \tag{S9}$$

In the LFOR, the wall loss rate is very low. For example, we can get a ~100% yield of semi-volatile sulfuric acid aerosol in the LFOR [6]. Therefore, we assume that the $k_{cond,w}$ in the LFOR is half of that in the smog chamber, which is 8.5 × 10^{-4} s^{-1}. The $k_{evap,w}$ in the LFOR is also calculated with Eq. (S8).

The other parameters used in the model are as follows: pressure 1 bar, temperature 298 K, RH 50%, duration time 3 h for the smog chamber and 160 s for the LFOR. When the model is running, the aerosol concentration and *CS* are calculated in real-time. For simplification, particle wall loss is not considered in the model. With this KinSim kinetic integrator, we can model the concentrations of LVOC and SVOC that get lost to the wall, condense on the aerosols, and stay in the gas phase during the residence time in the smog chamber and in the LFOR, which are shown in Fig. S9.

S5.2 LVOC, SVOC, and IVOC fates considering OH reactions

The above model only considers the condensation and evaporation of LVOC and SVOC, which shows that the wall loss is a large trap for SVOCs in smog chambers. However, the reactions with OH are also an important fate of organic vapors. Therefore, we further expand the model by: (1) considering OH reactions; (2) including IVOC; (3) adding a scenario of polluted ambient air.

(1) The OH exposure is set to 2.59 × 10^{11} molecules cm^{-3} s for all scenarios, which corresponds to an equivalent photochemical age of ~2 days (same age as the SOA composition data shown in Fig. 3a). The reaction rate constants with OH (k_{OH}) of IVOC is estimated as 2.65 × 10^{-11} cm^3 molecule^{-1} s^{-1} (see Text S6). We assume that IVOC is converted to SVOC after two steps of OH reactions, while SVOC is converted to LVOC after two steps of OH reactions based on the volatility change when adding functional groups [29]. The average carbon number and average oxygen number of IVOCs measured in this study are 6.4 and 1.9. Based on this, the number of oxygen atoms is assumed to be 3.9 for SVOC and 5.9 for LVOC, which are 2 and 4 oxygen atoms more than LVOC. We assume that the carbon number of SVOC and LVOC is the same as IVOC. We then calculated the k_{OH} of SVOC and LVOC based on the carbon and oxygen numbers using the equation from Donahue et al. [14]:

$$k_{OH} = 1.2\times10^{-12}\,(n_C + 9n_O - 10(n_O{:}n_C)^2) \tag{S10}$$

where n_C and n_O are carbon number and oxygen number, respectively. The k_{OH} of SVOC and LVOC are calculated as 4.4 × 10^{-11} cm^3 molecule^{-1} s^{-1} and 6.1 × 10^{-11} cm^3 molecule^{-1} s^{-1}, respectively, which are similar to the results estimated previously [14].

(2) For IVOC, the initial concentration is also set as 1 ppb. The $k_{cond,p}$ and $k_{evap,p}$ are calculated using the same method as SVOC shown in Text S5.1 It is found that the condensation rate of IVOC is much lower than SVOC from our measurement and according to previous studies [27], thus the $k_{cond,w}$ of IVOC in smog chamber is assumed to be 10 times lower than that of SVOC (i.e., 1.7×10^{-4} s^{-1}). The $k_{evap,w}$ of IVOC in smog chamber is calculated using Eq. (S8) and Eq. (S9). For LFOR, the $k_{cond,w}$ and $k_{evap,w}$ are assumed to be half of those in smog chamber, which is the same assumption as SVOC.

(3) For the polluted ambient air, the $k_{cond,w}$ and $k_{evap,w}$ are set to zero for all organic vapors since there are no wall losses. The duration time is 2 days while the OH concentration is set as the global average value of 1.5×10^6 molecules cm^{-3}. All parameters are summarized in Table S5.

In addition to the two condensational fates (i.e., particle and wall), the third fate of OH reaction is added. For IVOC and SVOC, it is assumed that two steps of OH reactions would change the product category to lower volatility compounds. Therefore, the lifetime against OH reaction is calculated as 2 / (k_{OH} × [OH]).

For LVOC, a few steps of oxidation may lead to the formation of ELVOC but the main fate of ELVOC is highly similar to that of LVOC, i.e., condensation into particles. More steps of OH oxidation may lead to fragmentation that forms higher volatility compounds that can stay in the gas phase. Therefore, we assume that 5 steps of oxidation are required for the fragmentation, same as a previous study [22]. This leads to the lifetime against OH reaction for LVOC to be 5 / (k_{OH} × [OH]). The model results considering OH reactions are shown in Fig. S10.

Text S6. IVOC SOA yields estimation

Using the concentration, chemical information, and estimated SOA production of the IVOCs (~70% of total SOA in LFOR experiments), we can estimate their average SOA yield. From the molecular formula and their relative intensities, the average molecular weight of 120 ± 24 Da is determined for these IVOCs. The reaction rate constants with OH (k_{OH}) are taken from literature when available (e.g., cresol and naphthalene) and are calculated based on molecular formula [14] for other NMOGs. The average k_{OH} is estimated to be $2.65 \pm 1.06 \times 10^{-11}$ cm^3 $molecule^{-1}$ s^{-1}. The average SOA yield of IVOCs was then calculated as dividing the 70% SOA mass concentration by the reacted IVOC mass concentration.

Text S7. Potential OFR limitations

The high radical (including OH, HO_2, etc.) concentrations and the UV light source for OFRs have the potential to lead to chemistry in the OFR which may be less relevant to the troposphere. A series of modeling studies have addressed these issues recently [30], indicating that reactions in OFRs can often be similar to ambient conditions when atmospheric oxidation is dominated by OH. The possible tropospherically-irrelevant/undesired reactions under some conditions are mainly considered to be from OH suppression caused by operating experiments with extremely high precursor concentrations (i.e., external OH reactivity, OHR_{ext}), an overly high UV photon flux and/or minimal humidity [30]. To prevent these issues in this study, we performed the LFOR experiments following the recommendations of previous studies [30-32]: high humidity (RH = ~50%), low ozone concentration (4 ppm), relatively low precursor concentration (OHR_{ext} < 30 s^{-1}), and relatively long residence time (~160 s).

A potential issue associated with OFRs is that the OH/HO_2 ratio at higher photochemical ages is likely to be higher than that of the typical ambient atmosphere, leading to a higher contribution from the RO_2+OH reaction to the fate of RO_2 [30, 32]. Another potential issue is that the RO_2 lifetime in the OFR might be shorter than in ambient air (due to higher radical concentrations), leading to insufficient time for RO_2 isomerization [30, 32]. These are common issues for all OFRs at higher photochemical ages and may lead to less relevant OFR chemistry/conditions, which represents the most significant limitation of OFRs.

Text S8. Sensitivity to SOA yield of $n_C \geq 6$ compounds

As shown in Text S2, we used a SOA yield of 0.32 for the $n_C \geq 6$ compounds with no reported yield data. However, NO_x condition and OA mass concentration influence the SOA yield. In a previous parameterization study [33], the SOA yield of $n_C \geq 6$ compounds in wood-burning emissions was determined in the range of 0.24−0.4 for OA mass concentration of 20−200 μg m^{-3} (the OA mass concentration range in this study). Therefore, we tested the influence of using lower and higher limits of the SOA yield (i.e., 0.24 and 0.40) on the SOA closure. As shown in Fig. S12a and 12b, the lower and

higher limits of the SOA yield lead to the explained SOA fractions of 24% and 30% for LFOR experiments, which are close to the fraction (27%) with a yield of 0.32. For SC experiments, the variations in SOA yield also influence little to the SOA closure results. This indicates that the SOA closure results are not highly sensitive to the SOA yield of $n_C \geq 6$ compounds.

Supplementary Figures and Tables

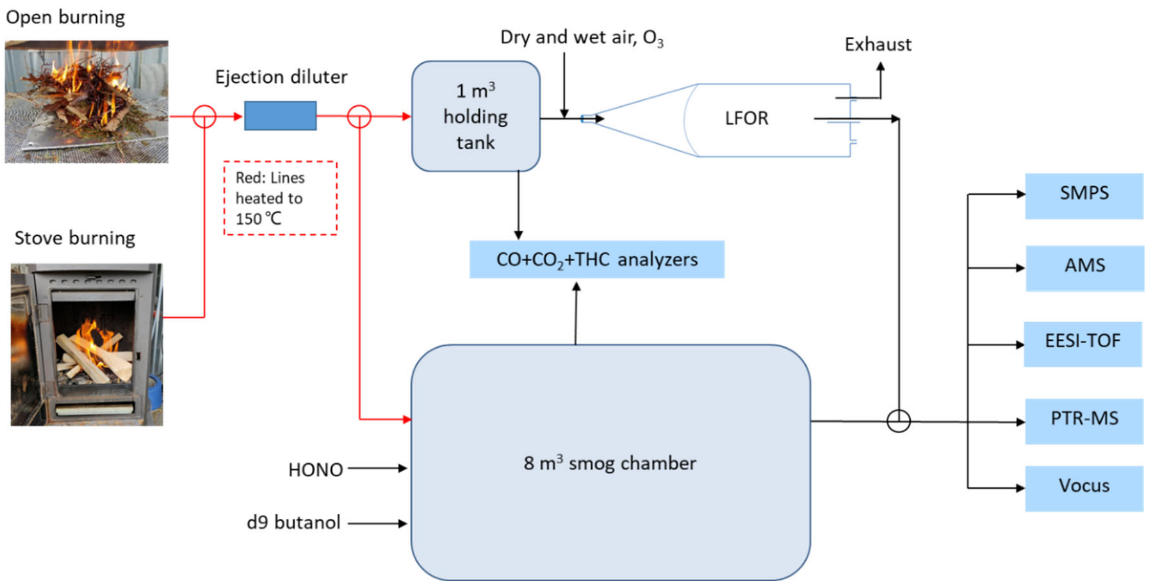

Figure S1. Experimental setup of the LFOR and smog chamber experiments of wood burning.

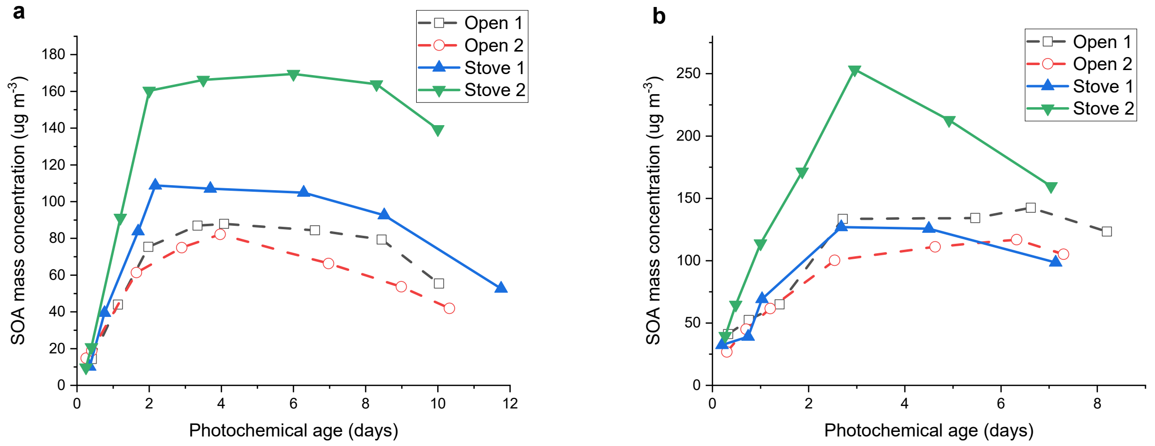

Figure S2. SOA production from open and stove burning. (a) Low-NO_x condition. (b) High-NO_x condition.

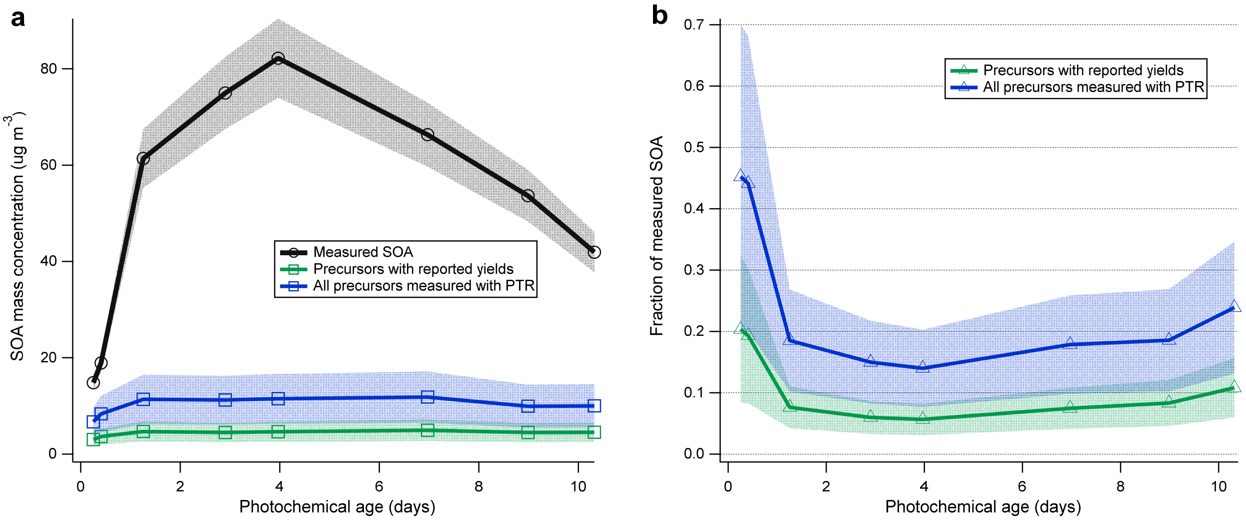

Figure S3. SOA production (a) and contribution (b) from NMOGs measured with PTR-MS in the open-low-2 experiment (before SC-LFOR correction).

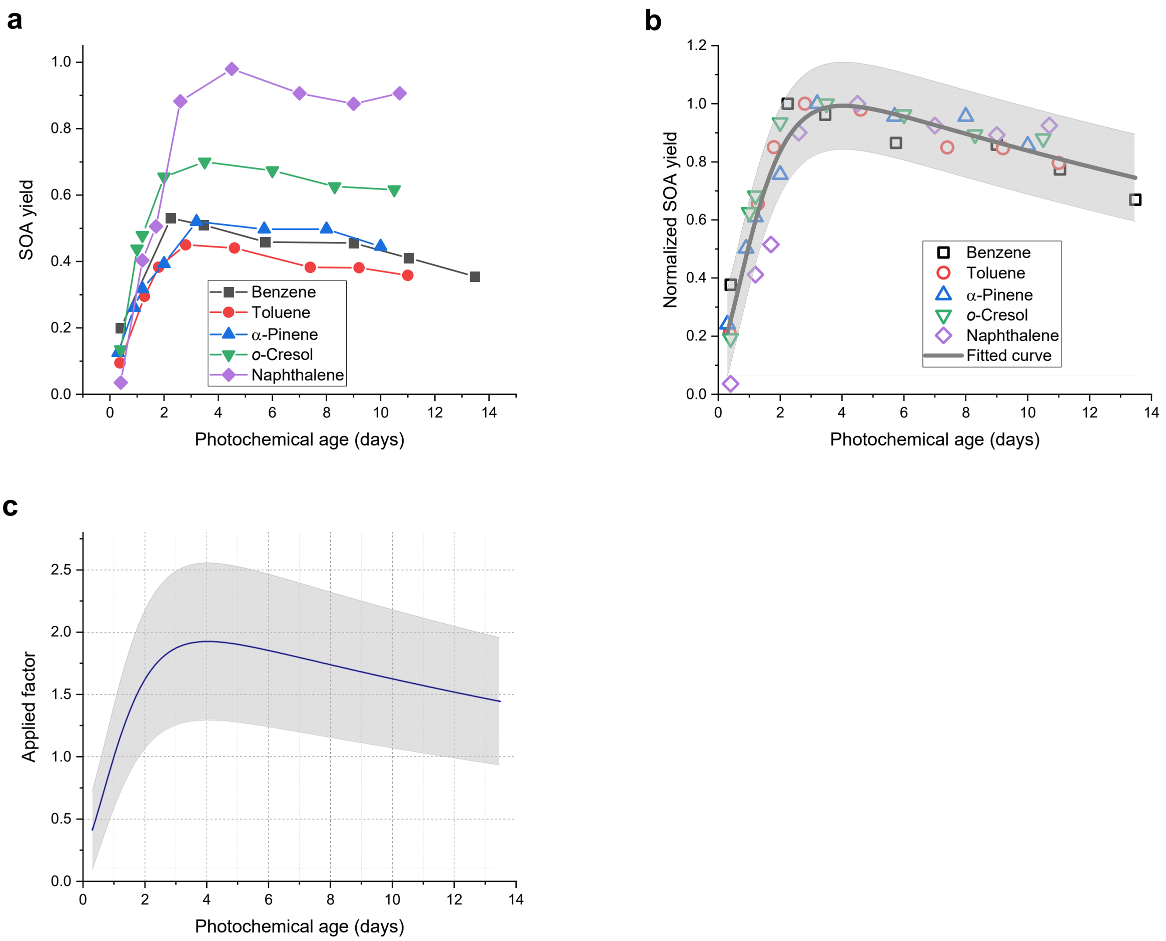

Figure S4. SC-LFOR SOA yield correction. (a) SOA yields of benzene, toluene, *α*-pinene, *o*-cresol, and naphthalene in the LFOR experiments. (b) Normalized SOA yields of the 5 single-compound LFOR experiments and the overall fitted curve. (c) The factor applied to the correction.

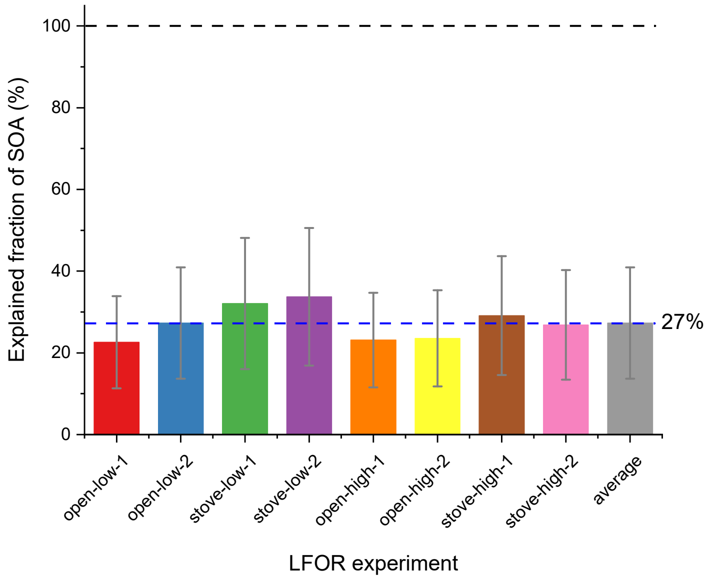

Figure S5. Explained fraction of SOA from the precursors measured by PTR-MS in the LFOR experiments. The black and blue dashed lines indicate 100% and 27%, respectively.

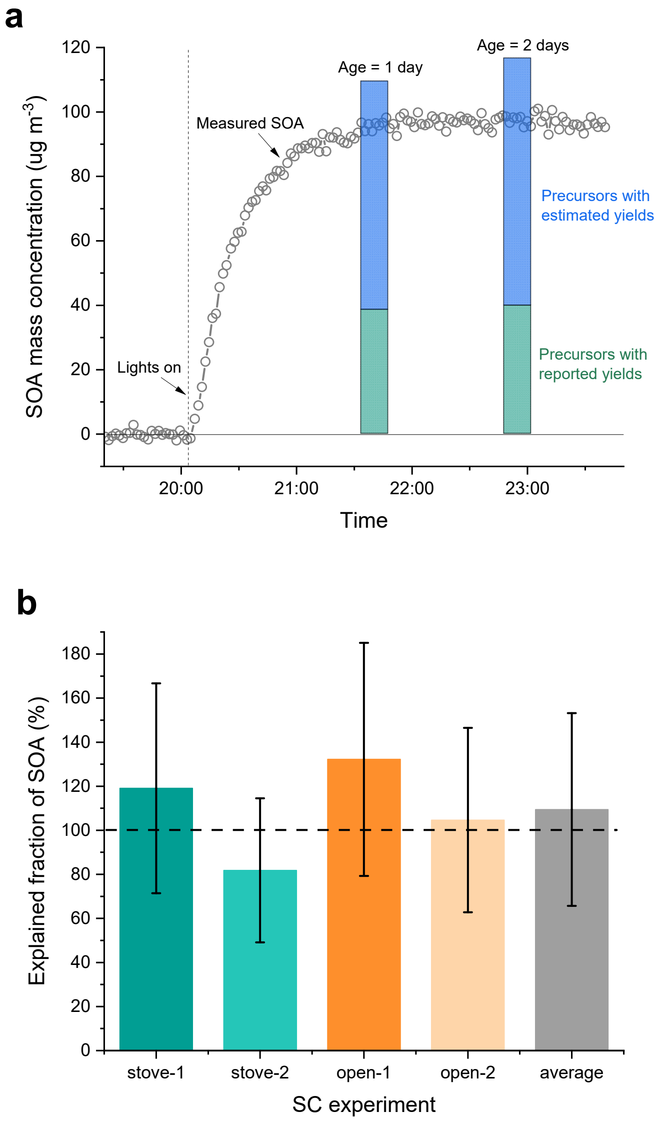

Figure S6. SOA closure from the smog chamber experiments. (a) Measured SOA time series and calculated SOA production from precursors measured with PTR-MS (experiment stove-SC-1 as an example). The SOA mass concentration was wall-loss corrected. The calculated SOA production is shown at photochemical ages of 1 day and 2 days. (b) Overall result at photochemical age of 1.5-2 days for all the smog chamber experiments.

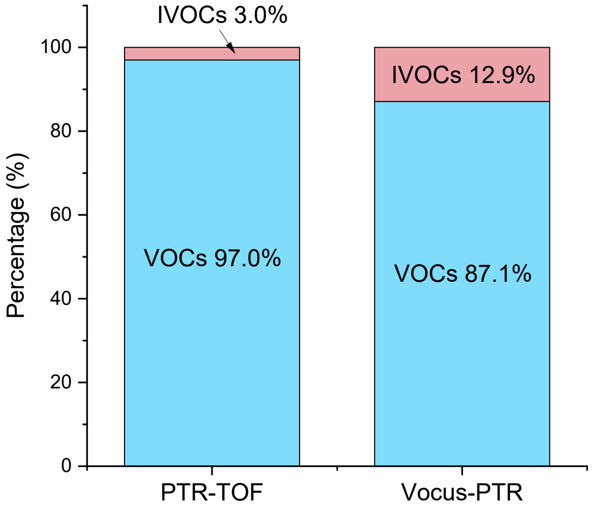

Figure S7. VOCs and IVOCs contribution to NMOGs measured with PTR-MS and Vocus-PTR for the LFOR experiments. For the SC experiments, the IVOC contribution is very similar (with a difference of <0.3% for PTR-MS and <2% for Vocus-PTR).

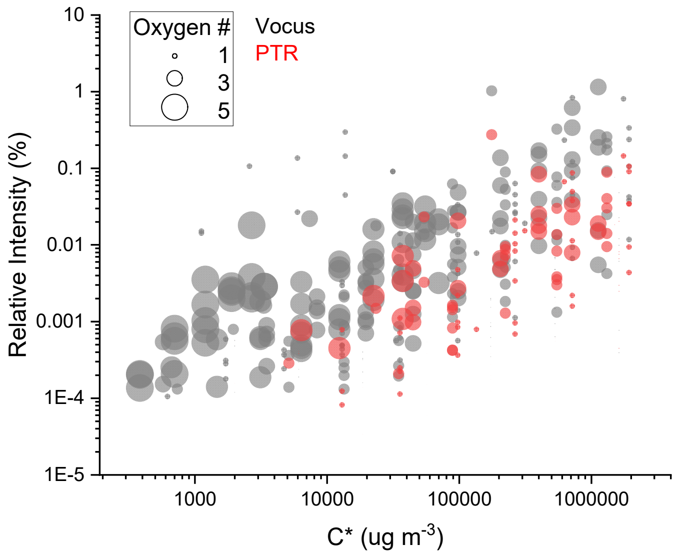

Figure S8. IVOC relative intensity measured with Vocus-PTR and PTR-MS. The size of the plot indicates the oxygen number in the molecule.

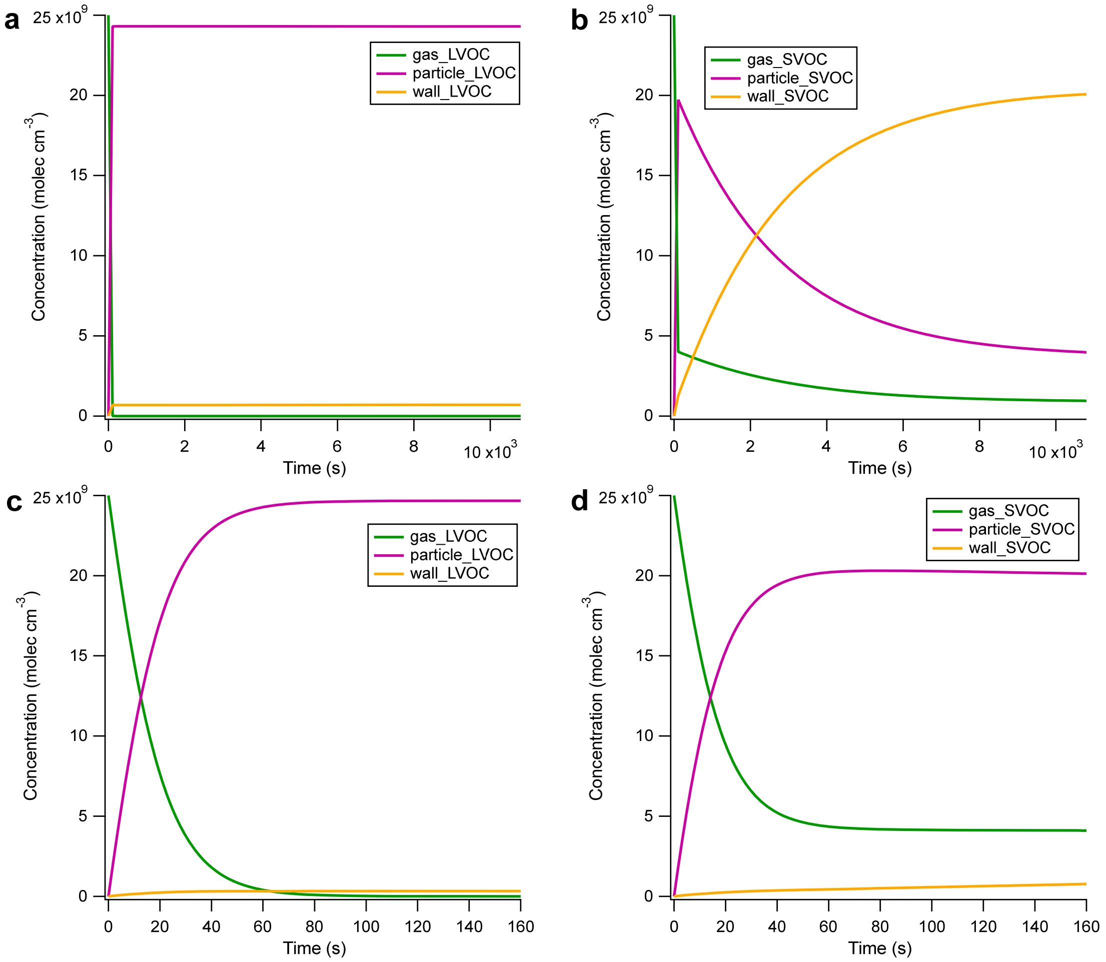

Figure S9. Modeled time series of LVOC and SVOC without considering reactions in the smog chamber (a, b) and in the LFOR (c, d).

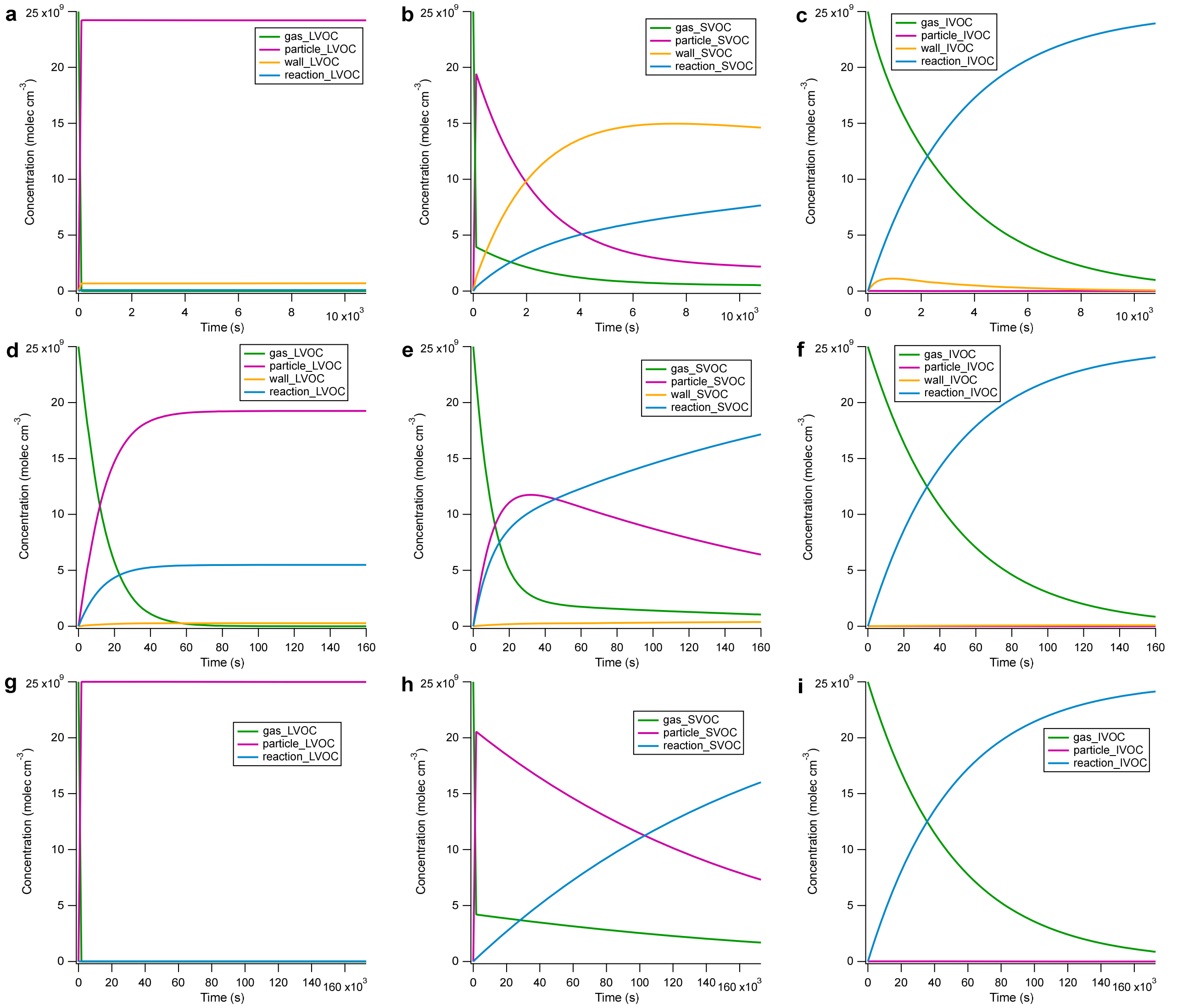

Figure S10. Modeled time series of LVOC, SVOC, and IVOC in the smog chamber (a, b, c), in the LFOR (d, e, f), and in the ambient air (g, h, i). Different from Fig. S9, the reactions with OH are considered in the simulations here.

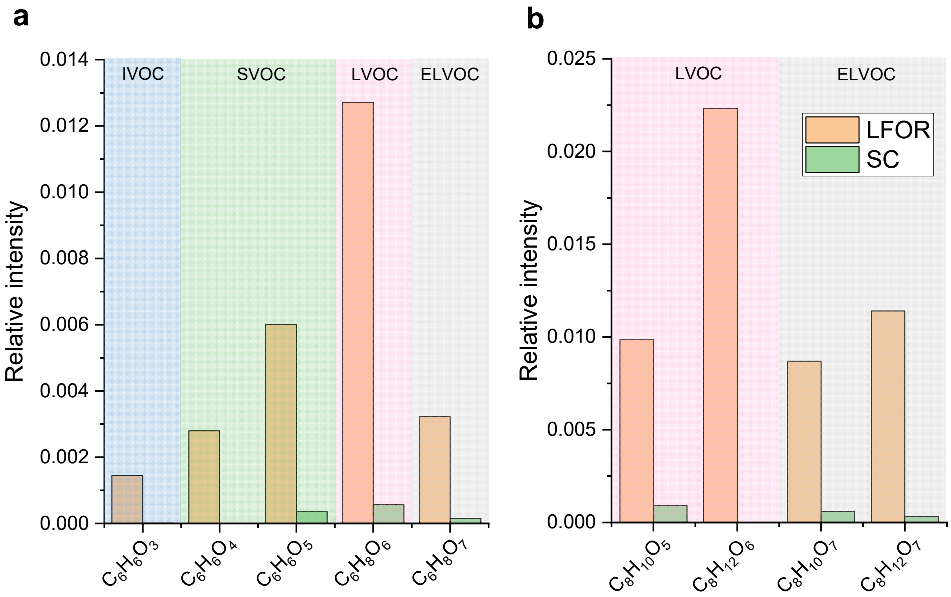

Figure S11. Relative intensities of representative products from the oxidation of (a) phenol and (b) dimethylphenol.

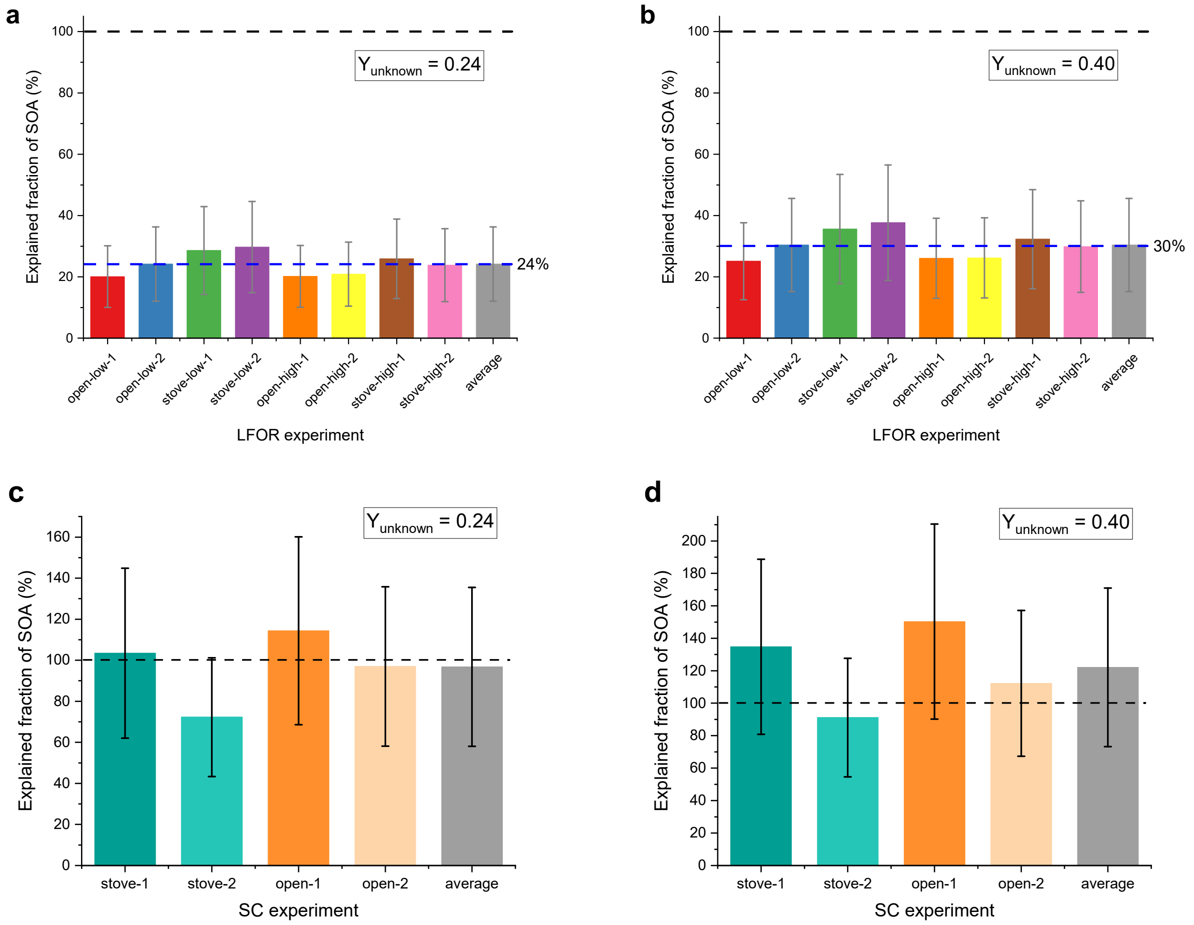

Figure S12. Explained fraction of SOA from the precursors measured by PTR-MS using different SOA yields for $n_C \geq 6$ compounds. (a, b) LFOR experiments with an SOA yield of 0.24 and 0.40 for $n_C \geq 6$ compounds. The blue lines show the average contribution of 24% and 30%, respectively. (c, d) SC experiments with an SOA yield of 0.24 and 0.40 for $n_C \geq 6$ compounds.

Table S1. Summary of the LFOR experiments for biomass-burning emissions.

Exp. No.	Burning type	NO_x condition	MCE	Emission factors (g kg^{-1})				
				CO	CO_2	THC	Primary PM	SOA-max
Open-low-1	open	low	0.95	54.20	1707.86	9.09	5.44	7.96
Open-low-2	open	low	0.91	64.61	1658.55	16.20	10.35	12.01
Stove-low-1	stove	low	0.90	56.68	1698.26	10.33	6.29	8.15
Stove-low-2	stove	low	0.92	71.90	1661.05	15.89	4.33	9.59
Open-high-1	open	high	0.91	70.53	1647.62	15.88	11.57	13.95
Open-high-2	open	high	0.93	64.58	1661.51	15.27	10.36	10.32
Stove-high-1	stove	high	0.90	98.26	1618.43	17.47	2.59	5.38
Stove-high-2	stove	high	0.94	66.43	1678.10	12.68	5.10	11.09

Table S2. Smog chamber SOA yield data applied to the calculation.

Name	PTR ion	m/z	Yield range	Average yield	Reference
Phenol	$C_6H_7O^+$	95.05	0.24-0.54	0.44	[34]
Naphthalene	$C_{10}H_9^+$	129.07	0.30-0.74	0.52	[35, 36]
Benzene	$C_6H_7^+$	79.05	0.28-0.37	0.33	[37]
o-Benzenediol	$C_6H_7O_2^+$	111.04	-	0.39	[38]
m-/o-Cresol	$C_7H_9O^+$	109.07	0.21-0.49	0.36	[38]
2-Methoxyphenol	$C_7H_9O2^+$	125.06	0.34-0.53	0.45	[34, 36]
2,4-/2,6-/3,5-Dimethylphenol	$C_8H_{11}O^+$	123.08	0.13-0.90	0.44	[38]
Toluene	$C_7H_9^+$	93.07	0.12-0.44	0.24	[37, 39]
2-/3-Methylfuran	$C_5H_7O^+$	83.05	0.055-0.085	0.07	[40]
1-/2-Methylnaphthalene	$C_{11}H_{11}^+$	143.09	0.38-0.71	0.52	[35]
Furan	$C_4H_5O^+$	69.03	0.019-0.072	0.05	[40]
Prop-2-enal	$C_3H_5O^+$	57.03	0.006-0.023	0.02	[36, 41]
2-Methylprop-2-enal/(2E)-2-butenal	$C_4H_7O^+$	71.05	0.013-0.052	0.03	[36, 41]
m-Xylene	$C_8H_{11}^+$	107.09	0.06-0.26	0.20	[36, 37]
1,2-Dimethylnaphthalene	$C_{12}H_{13}^+$	157.10	0.30-0.31	0.31	[35]
1,2-Dihydroacenaphthylene	$C_{12}H_{11}^+$	155.09	0.04-0.13	0.07	[42]
Isoprene	$C_5H_9^+$	69.07	0.02-0.06	0.04	[43]
Monoterpenes	$C_{10}H_{17}^+$	137.13	0.26-0.46	0.36	[44, 45]
2,4-/2,5-Dimethylfuran	$C_6H_9O^+$	97.07	-	0.32[a]	-
Styrene	$C_8H_9^+$	105.07	-	0.32[a]	-
Benzaldehyde	$C_7H_7O^+$	107.05	-	0.32[a]	-
4-(2-Hydroxyethyl)phenol/2-methoxy-4-methylphenol	$C_8H_{11}O_2^+$	139.08	-	0.32[a]	-
Other $n_C \geq 6$ compounds	-	-	-	0.32[a]	-

a. Average of applied yields from NMOGs with carbon number ≥ 6 [12].

Table S3. Summary of the LFOR experiments for single NMOGs.

NMOG name	NMOG conc. (ppb)	Max SOA mass (μg m^{-3})	Max yield
Benzene	47.3	79.8	0.53
Toluene	31.4	53.2	0.45
α-Pinene	26.5	77.0	0.52
o-Cresol	19.7	60.9	0.70
Naphthalene	10.9	56.0	0.98

Table S4. Summary of the smog chamber experiments.

**Exp. No.**	**Burning type**	**MCE**	**Emission factors (g kg^{-1})**				
			**CO**	**CO_2**	**THC**	**Primary PM**	**SOA-max**
Stove-SC-1	stove	0.96	44.43	1728.84	9.52	2.20	3.63
Stove-SC-2	stove	0.96	40.58	1716.79	16.01	1.16	3.89
Open-SC-1	open	0.90	110.84	1567.56	26.60	3.71	4.75
Open-SC-2	open	0.88	133.21	1471.01	45.03	5.35	3.78

Table S5. Parameters for IVOC, SVOC, and LVOC applied in the kinetic model.

Parameter	IVOC[b]	SVOC	LVOC
	**General**		
C* (μg m^{-3})	100000	10	0.001
Initial C_{OA} (μg m^{-3})	30	30	30
Concentration (ppb)	1	1	1
$k_{cond,p}$		Calculated real time[a]	
$k_{evap,p}$	$k_{cond,p} \times C^* / C_{OA}$	$k_{cond,p} \times C^* / C_{OA}$	0
OH_{exp} (molecules cm^{-3} s)[b]		2.59×10^{11}	
k_{OH} (cm^3 $molecule^{-1}$ s^{-1})[b]	2.65×10^{-11}	4.4×10^{-11}	6.1×10^{-11}
	**Smog chamber**		
$k_{cond,w}$ (s^{-1})	1.7×10^{-4}	1.7×10^{-3}	1.7×10^{-3}
$k_{evap,w}$ (s^{-1})	1.0×10^{-3}	2.7×10^{-5}	0
Duration time		3 h	
	**LFOR**		
$k_{cond,w}$ (s^{-1})	8.5×10^{-5}	8.5×10^{-4}	8.5×10^{-4}
$k_{evap,w}$ (s^{-1})	5.0×10^{-4}	1.4×10^{-5}	0
Duration time		160 s	
	**Ambient**[b]		
$k_{cond,w}$ (s^{-1})		0	
$k_{evap,w}$ (s^{-1})		0	
Duration time		2 days	

a. See Text S5.1 for the calculation details.
b. Only included in the model of Text S5.2.

References

1. Andreae MO, Merlet P. Emission of trace gases and aerosols from biomass burning. *Global Biogeochem Cycles* 2001; **15**(4): 955-966. doi: 10.1029/2000gb001382
2. Bertrand A, Stefenelli G, Bruns EA *et al.* Primary emissions and secondary aerosol production potential from woodstoves for residential heating: Influence of the stove technology and combustion efficiency. *Atmos Environ* 2017; **169**: 65-79. doi: 10.1016/j.atmosenv.2017.09.005
3. Bruns EA, Krapf M, Orasche J *et al.* Characterization of primary and secondary wood combustion products generated under different burner loads. *Atmos Chem Phys* 2015; **15**(5): 2825-2841. doi: 10.5194/acp-15-2825-2015
4. Li K, Wentzell JJB, Liu Q *et al.* Evolution of Atmospheric Total Organic Carbon from Petrochemical Mixtures. *Environ Sci Technol* 2021; **55**(19): 12841-12851. doi: 10.1021/acs.est.1c02620
5. Li K, Liggio J, Han C *et al.* Understanding the Impact of High-NOx Conditions on the Formation of Secondary Organic Aerosol in the Photooxidation of Oil Sand-Related Precursors. *Environ Sci Technol* 2019; **53**(24): 14420-14429. doi: 10.1021/acs.est.9b05404
6. Li K, Liggio J, Lee P *et al.* Secondary organic aerosol formation from α-pinene, alkanes and oil-sands-related precursors in a new oxidation flow reactor. *Atmos Chem Phys* 2019; **19**(15): 9715-9731. doi: 10.5194/acp-19-9715-2019
7. Lambe A, Massoli P, Zhang X *et al.* Controlled nitric oxide production via O(1D)+N2O reactions for use in oxidation flow reactor studies. *Atmos Meas Tech* 2017; **10**(6): 2283-2298. doi: 10.5194/amt-10-2283-2017
8. Peng Z, Palm BB, Day DA *et al.* Model Evaluation of New Techniques for Maintaining High-NO Conditions in Oxidation Flow Reactors for the Study of OH-Initiated Atmospheric Chemistry. *ACS Earth Space Chem* 2018; **2**(2): 72-86. doi: 10.1021/acsearthspacechem.7b00070
9. Platt SM, El Haddad I, Zardini AA *et al.* Secondary organic aerosol formation from gasoline vehicle emissions in a new mobile environmental reaction chamber. *Atmos Chem Phys* 2013; **13**(18): 9141-9158. doi: 10.5194/acp-13-9141-2013
10. Barmet P, Dommen J, DeCarlo PF *et al.* OH clock determination by proton transfer reaction mass spectrometry at an environmental chamber. *Atmos Meas Tech* 2012; **5**(3): 647-656. doi: 10.5194/amt-5-647-2012
11. Mao J, Ren X, Brune WH *et al.* Airborne measurement of OH reactivity during INTEX-B. *Atmos Chem Phys* 2009; **9**(1): 163-173.
12. Bruns EA, El Haddad I, Slowik JG *et al.* Identification of significant precursor gases of secondary organic aerosols from residential wood combustion. *Sci Rep* 2016; **6**: 27881. doi: 10.1038/srep27881
13. Lopez-Hilfiker FD, Pospisilova V, Huang W *et al.* An extractive electrospray ionization time-of-flight mass spectrometer (EESI-TOF) for online measurement of atmospheric aerosol particles. *Atmos Meas Tech* 2019; **12**(9): 4867-4886. doi: 10.5194/amt-12-4867-2019
14. Donahue NM, Chuang W, Epstein SA *et al.* Why do organic aerosols exist? Understanding aerosol lifetimes using the two-dimensional volatility basis set. *Environ Chem* 2013; **10**(3): 151-157. doi: 10.1071/en13022
15. Koss AR, Sekimoto K, Gilman JB *et al.* Non-methane organic gas emissions from biomass burning: identification, quantification, and emission factors from PTR-ToF during the FIREX 2016 laboratory experiment. *Atmos Chem Phys* 2018; **18**(5): 3299-3319. doi: 10.5194/acp-18-3299-2018
16. Li Y, Pöschl U, Shiraiwa M. Molecular corridors and parameterizations of volatility in the chemical evolution of organic aerosols. *Atmos Chem Phys* 2016; **16**(5): 3327-3344. doi: 10.5194/acp-16-3327-2016
17. Cappellin L, Karl T, Probst M *et al.* On quantitative determination of volatile organic compound concentrations using proton transfer reaction time-of-flight mass spectrometry. *Environ Sci Technol* 2012; **46**(4): 2283-2290. doi: 10.1021/es203985t
18. Lambe AT, Chhabra PS, Onasch TB *et al.* Effect of oxidant concentration, exposure time, and seed particles on secondary organic aerosol chemical composition and yield. *Atmos Chem Phys* 2015; **15**(6): 3063-3075. doi: 10.5194/acp-15-3063-2015
19. Isaacman-VanWertz G, Massoli P, O'Brien R *et al.* Chemical evolution of atmospheric organic carbon over multiple generations of oxidation. *Nat Chem* 2018; **10**: 462-468. doi: 10.1038/s41557-018-0002-2
20. Peng Z, Jimenez JL. KinSim: A Research-Grade, User-Friendly, Visual Kinetics Simulator for Chemical-Kinetics and Environmental-Chemistry Teaching. *J Chem Educ* 2019; **96**(4): 806-811. doi: 10.1021/acs.jchemed.9b00033
21. Krechmer JE, Day DA, Jimenez JL. Always Lost but Never Forgotten: Gas-Phase Wall Losses Are Important in All Teflon Environmental Chambers. *Environ Sci Technol* 2020; **54**(20): 12890-12897. doi: 10.1021/acs.est.0c03381
22. Palm BB, Campuzano-Jost P, Ortega AM *et al.* In situ secondary organic aerosol formation from ambient pine forest air using an oxidation flow reactor. *Atmos Chem Phys* 2016; **16**(5): 2943-2970. doi: 10.5194/acp-16-2943-2016
23. Tang MJ, Shiraiwa M, Pöschl U *et al.* Compilation and evaluation of gas phase diffusion coefficients of reactive trace gases in the atmosphere: Volume 2. Diffusivities of organic compounds, pressure-normalised mean free paths, and average Knudsen numbers for gas uptake calculations. *Atmos Chem Phys* 2015; **15**(10): 5585-5598. doi: 10.5194/acp-15-5585-2015

24. Seinfeld JH, Pandis SN. *Atmospheric chemistry and physics: from air pollution to climate change*. Hoboken: John Wiley & Sons, 2016.

25. Krechmer JE, Day DA, Ziemann PJ *et al.* Direct Measurements of Gas/Particle Partitioning and Mass Accommodation Coefficients in Environmental Chambers. *Environ Sci Technol* 2017; **51**(20): 11867-11875. doi: 10.1021/acs.est.7b02144

26. Bertrand A, Stefenelli G, Pieber SM *et al.* Influence of the vapor wall loss on the degradation rate constants in chamber experiments of levoglucosan and other biomass burning markers. *Atmos Chem Phys* 2018; **18**(15): 10915-10930. doi: 10.5194/acp-18-10915-2018

27. Krechmer JE, Pagonis D, Ziemann PJ *et al.* Quantification of Gas-Wall Partitioning in Teflon Environmental Chambers Using Rapid Bursts of Low-Volatility Oxidized Species Generated in Situ. *Environ Sci Technol* 2016; **50**(11): 5757-5765. doi: 10.1021/acs.est.6b00606

28. Matsunaga A, Ziemann PJ. Gas-Wall Partitioning of Organic Compounds in a Teflon Film Chamber and Potential Effects on Reaction Product and Aerosol Yield Measurements. *Aerosol Sci Technol* 2010; **44**(10): 881-892. doi: 10.1080/02786826.2010.501044

29. Kroll JH, Seinfeld JH. Chemistry of secondary organic aerosol: Formation and evolution of low-volatility organics in the atmosphere. *Atmos Environ* 2008; **42**(16): 3593-3624. doi: 10.1016/j.atmosenv.2008.01.003

30. Peng Z, Jimenez JL. Radical chemistry in oxidation flow reactors for atmospheric chemistry research. *Chem Soc Rev* 2020; **49**(9): 2570-2616. doi: 10.1039/c9cs00766k

31. Peng Z, Day DA, Ortega AM *et al.* Non-OH chemistry in oxidation flow reactors for the study of atmospheric chemistry systematically examined by modeling. *Atmos Chem Phys* 2016; **16**(7): 4283-4305. doi: 10.5194/acp-16-4283-2016

32. Peng Z, Lee-Taylor J, Orlando JJ *et al.* Organic peroxy radical chemistry in oxidation flow reactors and environmental chambers and their atmospheric relevance. *Atmos Chem Phys* 2019; **19**(2): 813-834. doi: 10.5194/acp-19-813-2019

33. Stefenelli G, Jiang J, Bertrand A *et al.* Secondary organic aerosol formation from smoldering and flaming combustion of biomass: a box model parametrization based on volatility basis set. *Atmos Chem Phys* 2019; **19**(17): 11461-11484. doi: 10.5194/acp-19-11461-2019

34. Yee LD, Kautzman KE, Loza CL *et al.* Secondary organic aerosol formation from biomass burning intermediates: phenol and methoxyphenols. *Atmos Chem Phys* 2013; **13**(16): 8019-8043. doi: 10.5194/acp-13-8019-2013

35. Chan AWH, Kautzman KE, Chhabra PS *et al.* Secondary organic aerosol formation from photooxidation of naphthalene and alkylnaphthalenes: implications for oxidation of intermediate volatility organic compounds (IVOCs). *Atmos Chem Phys* 2009; **9**(9): 3049-3060. doi: 10.5194/acp-9-3049-2009

36. Chhabra PS, Ng NL, Canagaratna MR *et al.* Elemental composition and oxidation of chamber organic aerosol. *Atmos Chem Phys* 2011; **11**(17): 8827-8845. doi: 10.5194/acp-11-8827-2011

37. Ng NL, Kroll JH, Chan AWH *et al.* Secondary organic aerosol formation from m-xylene, toluene, and benzene. *Atmos Chem Phys* 2007; **7**(14): 3909-3922.

38. Nakao S, Clark C, Tang P *et al.* Secondary organic aerosol formation from phenolic compounds in the absence of NOx. *Atmos Chem Phys* 2011; **11**(20): 10649-10660. doi: 10.5194/acp-11-10649-2011

39. Hildebrandt L, Donahue NM, Pandis SN. High formation of secondary organic aerosol from the photo-oxidation of toluene. *Atmos Chem Phys* 2009; **9**(9): 2973-2986.

40. Gómez Alvarez E, Borrás E, Viidanoja J *et al.* Unsaturated dicarbonyl products from the OH-initiated photo-oxidation of furan, 2-methylfuran and 3-methylfuran. *Atmos Environ* 2009; **43**(9): 1603-1612. doi: 10.1016/j.atmosenv.2008.12.019

41. Chan AWH, Chan MN, Surratt JD *et al.* Role of aldehyde chemistry and NOx concentrations in secondary organic aerosol formation. *Atmos Chem Phys* 2010; **10**(15): 7169-7188. doi: 10.5194/acp-10-7169-2010

42. Shakya KM, Griffin RJ. Secondary Organic Aerosol from Photooxidation of Polycyclic Aromatic Hydrocarbons. *Environ Sci Technol* 2010; **44**(21): 8134-8139. doi: 10.1021/es1019417

43. Kroll JH, Ng NL, Murphy SM *et al.* Secondary organic aerosol formation from isoprene photooxidation. *Environ Sci Technol* 2006; **40**(6): 1869-1877. doi: 10.1021/es0524301

44. Eddingsaas NC, Loza CL, Yee LD *et al.* α-pinene photooxidation under controlled chemical conditions – Part 2: SOA yield and composition in low- and high-NOx environments. *Atmos Chem Phys* 2012; **12**(16): 7413-7427. doi: 10.5194/acp-12-7413-2012

45. Ng NL, Chhabra PS, Chan AWH *et al.* Effect of NOx level on secondary organic aerosol (SOA) formation from the photooxidation of terpenes. *Atmos Chem Phys* 2007; **7**(19): 5159-5174.
